# Evaluation of 11 commercially available PCR kits for the detection of monkeypox virus DNA, Berlin, July to September 2022 

**DOI:** 10.2807/1560-7917.ES.2022.27.45.2200816

**Published:** 2022-11-10

**Authors:** Janine Michel, Angelina Targosz, Thomas Rinner, Daniel Bourquain, Annika Brinkmann, Jilian Amber Sacks, Lars Schaade, Andreas Nitsche

**Affiliations:** 1 Robert Koch Institute (RKI), Center for Biological Threats and Special Pathogens, German Reference Laboratory for Poxviruses, Berlin, Germany; 2 World Health Organization (WHO), Department of Epidemic and Pandemic Preparedness and Prevention, Geneva, Switzerland

**Keywords:** monkeypox, orthopoxviruses, PCR, diagnostics

## Abstract

Before the international spread of monkeypox in May 2022, PCR kits for the detection of orthopoxviruses, and specifically monkeypox virus, were rarely available. Here we describe the evaluation of 11 recently developed commercially available PCR kits for the detection of monkeypox virus DNA. All tested kits are currently intended for research use only and clinical performance still needs to be assessed in more detail, but all were suitable for diagnostics of monkeypox virus, with variations in specificity rather than sensitivity.

Since May 2022, an increasing number of cases of human monkeypox have been noted worldwide, outside established endemic areas, particularly in Europe and the United States (US). The World Health Organization declared this outbreak a public health emergency of international concern on 23 July 2022 [1].

As is often the case for rare and neglected diseases, there is a lack of quality-assured tools to control monkeypox, including reliable, commercially available test kits for diagnosis. Until recently, few PCR kits were available, many of which were designed to detect *Orthopoxviruses* (OPV) and/or variola virus, the causative agent of smallpox. Hence, specialised laboratories relied on well validated, in-house protocols for diagnostics. The increased spread of human monkeypox has triggered the development of PCR kits designed to detect either monkeypox virus (MPXV) specifically or OPV – the genus comprising monkeypox virus (MPXV), vaccinia virus (VACV) and cowpox virus (CPXV), zoonotic viruses that can cause sporadic human infections or self-limiting outbreaks, but also variola virus. Since MPXV clinical samples have been rare in the past, kits are often validated by in silico comparison to published sequences.

The aim of this work was to evaluate commercially available PCR kits for the detection of MPXV DNA.

## Preparation of the evaluation panel

To evaluate ready-to-use kits for MPXV diagnostics, we established an 18-specimen panel using DNA from cultured viruses (Table 1) and characterised it using the diagnostic workflow of the German Consultant Laboratory for poxviruses (Table 2) [2-4]. The panel included DNA from MPXV Clade I, Clade IIa [5] and Clade IIb, other OPV and varicella zoster virus (VZV). All samples were analysed in duplicate.

**Table 1:** Composition of the evaluation panel for PCR kits developed to detect Orthopoxviruses or monkeypox virus

Cq aim	Monkeypox virus			Other orthopoxviruses
	Clade II 2012 [5]	Clade IIb 2022^a^	Clade I^b^	
17	Ni	Ni	Ni	VACV^c^, VZV^d^
19	Ni	Ni	Ni	CPXV^c^
28	X	X	Ni	Not included
29	Ni	Ni	X	
30	X	X	Ni	
32	X	X	X	
34	X	X	Ni	
35	Ni	Ni	X	
36	X	X	Ni	
38	X	X	Ni	

CPXV: cowpox virus; Cq: quantification cycle; Ni: not included; VACV: vaccinia virus; VZV: varicella zoster virus; X: included.

^a^ 2022, MPXV/Germany/2022/ON/RKI305, GenBank accession number: OP494258.

^b^ MSF6, kindly provided by Hermann Meyer, Bundeswehr Institute of Microbiology, Munich.

^c^ Cq determined with the rpo18 PCR [4].

^d^ Cq determined by in-house PCR.

**Table 2:** In-house protocol used as reference for detection of monkeypox virus DNA

Assay	Oligonucleotide name	Sequence
OPV generic (rpo18 gene)	rpo F	CTgTAgTTATAAACgTTCCgTgTg
	rpo R	TTATCATACgCATTACCATTTCgA
	rpo probe^a^	FAM-ATCgCTAAATgATACAgTACCCgAA T* CTCTACT p
KoMa internal control	KoMa F	ggTgATgCCgCATTATTACTAgg
	KoMa R	ggTATTAgCAgTCgCAggCTT
	KoMa probe	TEX-TTCTTgCTTgAggATCTgTCgTggATCg-BBQ
MPXV generic (G2R gene)	G2R_G F	ggAAAATgTAAAgACAACgAATACAg
	G2R_G R	gCTATCACATAATCTggAAgCgTA
	G2R_G probe	FAM-AAgCCgTAATCTATgTTgTCTATCgTgTCC-BHQ1
MPXV Clade II (G2R gene)	G2R_WA F	CACACCgTCTCTTCCACAgA
	G2R_WA R	gATACAggTTAATTTCCACATCg
	G2R_WA probe	FAM-AACCCgTCgTAACCAgCAATACATTT-BHQ1
*MYC* control	c-myc F	gCCAgAggAggAACgAgCT
	c-myc R	gggCCTTTTCATTgTTTTCCA
	c-myc probe	6FAM-TgCCCTgCgTgACCAgATCC-BHQ1

BBQ: blackberry quencher; BH: black hole quencher; BHQ1: black hole quencher 1; FAM: fluorescein; p: phosphate; MPXV: monkeypox virus; OPV: orthopoxvirus; TEX: Texas red.

^a^ T* marks the position of the quencher.

To ensure comparability of results between the different PCR kits, we prepared 500 µL of each sample in the panel by extraction of DNA from cell culture supernatant using the QIAamp DNA Blood Mini Kit and assessed the Cq values using the rpo18 PCR [4] or an in-house VZV PCR. DNA was pre-diluted to a calculated Cq of 28 (Clade II) or of 29 (Clade I) in lambda DNA (1 ng/μL; MBI Fermentas, Leon-Roth, Germany) and further diluted to obtain a calculated Cq of 38 (Clade II) or 35 (Clade I) in a fourfold or eightfold dilution series, respectively. All OPV samples were quantified using a plasmid standard, aliquoted to 50 µL and stored at −20 °C until use. Stability of the samples was confirmed through repeated use of the in-house MPXV PCR protocol (Table 2).

A standard PCR protocol was used for the reference PCRs, using 20 µL of master mix and 5 µL of DNA sample volume per reaction. The detailed protocols are appended in Supplementary Tables S1 and S2.

## Evaluation of commercially available PCR kits

We compared 11 kits (A to L) (Table 3) with the reference diagnostic workflow. This included one generic OPV PCR [4], one MPXV-specific PCR and one MPXV Clade II-specific PCR [2]. In addition, an inhibition control was spiked into the specimens before DNA extraction [6] and proper sampling was verified using a human DNA-specific PCR reaction [3]. We used all kits according to the manufacturer's manual and the threshold was set to obtain the lowest possible Cq value. For better comparability, we ran all kits on the BioRad CFX 96 real-time cycler, which is compatible with the fluorophores used by all included tests, even if it was not specifically noted in the manual. The results are summarised in the Figure.

**Table 3:** Characteristics of kits evaluated for monkeypox virus detection

Test	Manufacturer	Product name	Detectable virus (channel)	Gene region	Internal control (channel)	Sample volume/total volume (µL)	LOD according to manual
A	ACON Biotech	Promotor Monkeypox Virus Real Time PCR Test Kit	MPXV (FAM)	F3L MPXV	Exogenous IC (VIC)	5/25	250 copies/mL
B	Altona Diagnostics	Real Star Zoonotic Orthopoxvirus PCR kit 1.0	OPV (FAM)	NP	Heterologous IC (JOE)	10/30	NP
C	Bioperfectus Technologies	Monkeypox Virus Real Time PCR Kit	MPXV (FAM)	F3L MPXV	Endogenous IC RNAse P (VIC)	5/25	5 copies/rxn
D	DaAn Gene	Detection Kit for Monkeypox Virus DNA	MPXV (FAM)	F3L MPXV	Endogenous IC RNAse P (VIC)	10/30	200 copies/mL
E	Shanghai ZJ Bio-Tech Co., Ltd. (“Liferiver”)	Monkeypox Virus Real Time PCR Kit	MPXV (FAM)	F3L MPXV^a^	Exogenous IC (HEX/JOE/VIC)	4/40	5 × 10^3^ copies/ml
F	NOVACYT^b^	GenesigMonkeypox virus M3L gene	MPXV (FAM)	M3L MPXV	Endo-/exo-genous IC (FAM/VIC)	5/20	< 100 copies of target
G	Perkin Elmer	Pkamp Monkeypox Virus RT-PCR RUO Kit	MPXV (FAM)	F3L MPXV	Endogenous RNAse P IC (HEX/VIC)	10/15	20 copies/rxn
H	Sansure Biotech	Monkeypox virus Nucleic Acid Diagnostic Kit	MPXV (FAM)	F3L MPXV	Endogenous human gene IC (Cy5)	10/50	200 copies/mL
I	ThermoFisher	TaqMan Monkeypox Virus Microbe Detection Assay	MPXV (FAM)	J1L MPXV	None	9/20	NP
K	TIB Molbiol^c^	LightMix Modular Orthopox Virus	OPV (FAM)	14 kDa OPV	None	5/20	< 10 copies/rxn
L	TIB Molbiol^c^	LightMix Modular Monkeypox Virus	MPXV (HEX)	J2L/J2R MPXV	None	5/20	< 10 copies/rxn

FAM: fluorescein; HEX: hexachloro-fluorescein; IC: internal control; JOE: 5'-dichloro-dimethoxy-fluorescein; LOD: limit of detection; MPXV: monkeypox virus; NP: not provided; OPV: orthopoxvirus; rxn: reaction; VIC: 2′-chloro-7′phenyl-1,4-dichloro-6-carboxy-fluorescein.

^a^ A prior version of the kit targeted F2L and F3L, but the current kit only targets F3L.

^b^ Novacyt’s genesig MonkeyPox kit, evaluated in this study, is no longer available (as per end of September 2022); the company is redesigning an updated kit.

^c^ Used with the lyophilised 1-step RT-PCR polymerase mix.

**Figure:**
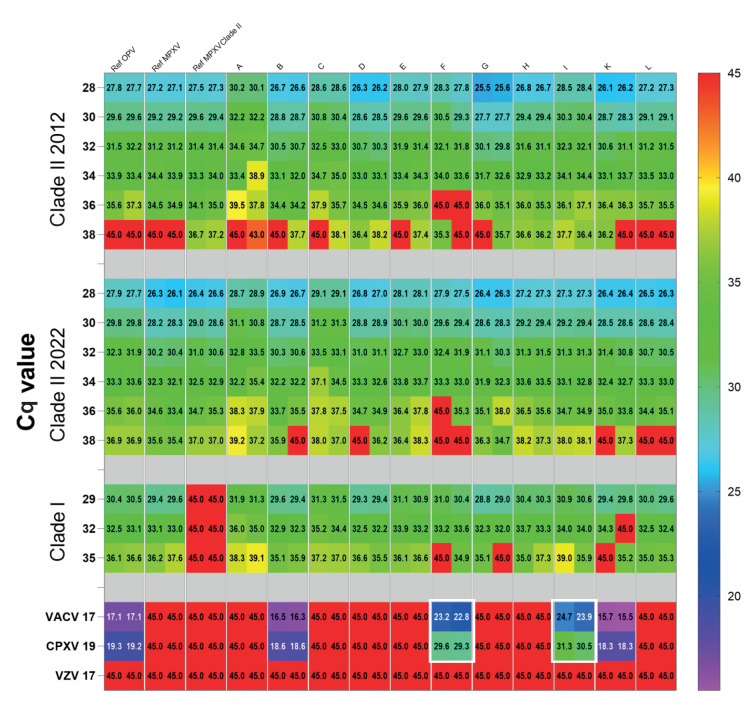
Summary of Cq values obtained for the evaluation panel with monkeypox virus PCR kits A to L

CPXV: cowpox virus; C_q_: cycle threshold; MPXV: monkeypox virus; OPV: orthopoxviruses; ref: reference PCR; VACV: vaccinia virus; VZV: varicella zoster virus.

A total of 45 cycles were run, so negative values are indicated in red boxes with Cq = 45. Coloured boxes reflect Cq values in the range 15–45. Bold white frames highlight cross-reactivity of kits F and I with VACV and CPXV. Cq values presented in the three columns on the left were obtained with the reference PCRs (rpo18 for OPV [4], and in-house PCR for VZV).

## Analytical sensitivity of the kits

For the 2012 Clade II MPXV isolate, most kits showed Cq values in the expected range, indicating good analytical sensitivity down to at least Cq = 36, reflecting approximately 5 genome equivalents (ge) per reaction (rxn). Only kit F failed to detect this dilution. For the highest dilution (Cq = 38, reflecting < 1 ge/rxn), two kits and two generic reference PCRs for OPV and MPXV failed to detect both replicates; six kits detected one of two replicates; three kits and the Clade II-specific reference PCR detected both replicates, indicating high analytical sensitivity. It should be noted that in this range of DNA concentration, results are prone to higher statistical variation compared than at higher concentrations, and that it may not necessarily reflect poor test performance if only one of the two technical duplicate reactions gave a signal. Using a 2022 Clade IIb instead of a 2012 MPXV isolate (II), the results were similar, with slightly better detection rates of samples with higher Cq. Only kits F and L failed to detect the lowest virus load (Cq = 38), while three kits only detected one of the duplicate reactions. 

We also assessed Clade I MPXV detection: kit K failed to detect one duplicate of the sample with Cq = 32, while three kits failed to detect one duplicate of the lowest concentration (Cq = 35). Similar Cq values were obtained across the different kits for most samples, particularly when considering the varying volumes used per reaction which may contribute to a shift in Cq values of approximately two to three cycles.

Importantly, all controls included in the kits performed as expected. All kits performed within the range specified by the respective manuals.

## Specificity testing of commercially available MPXV PCR kits

We also assessed specificity using VACV, CPXV and VZV DNA. As expected, none of the kits detected VZV (Figure). According to the manuals, all kits were designed to be specific for MPXV DNA, excluding other OPV, except for kits B and K which are generic for OPV (Table 3). The Figure confirms that the OPV kits B and K also detect VACV and CPXV DNA with Cq values similar to those of the OPV reference PCR [4] for the same samples whereas the MPXV-specific kits do not, with two exceptions: kits F and I unexpectedly detected VACV and CPXV DNA, indicating non-specific interactions. We also confirmed the results for kit I on the QuantStudio 5, the thermal cycler specifically recommended by the manufacturer. Both kits F and I resulted in similar Cq values, with Cq value shifts of ca six to seven cycles for VACV and 11–12 cycles for CPXV, indicating better, but still inefficient, binding to VACV DNA than to CPXV DNA. Although details on the primer and probe sequences were not provided by the manufacturers, kit F targets the M3L gene and kit I targets J1L. A sequence comparison of the M3L MPXV gene with 92 orthologues of CPXV and 109 VACV showed similarities of 94.6% to 96.8%, and for J1L, comparison of the MPXV gene with 80 orthologues of CPXV and 99 VACV showed similarities of 83.9% to 97.3%.

For further characterisation, we plotted the Cq values for each DNA sample compared with the calculated number of ge, determined by a plasmid standard curve [7] and determined the slope, reflecting PCR efficiency (ideally ca −3.32 assuming doubling of PCR product per cycle), and the Y-intercept, indicating the theoretically smallest positive Cq value obtained with an assay (Table 4). The curves are similar for all three virus strains. Details on this are provided as additional information in Supplementary Figure S1.

**Table 4:** Description of standard curves obtained from dilutions of monkeypox virus DNA with different PCR kits

	Ref OPV	Ref MPXV	Ref MPXV Clade II	Test kit										
				A	B	C	D	E	F	G	H	I	K	L
**Clade II 2012**														
Slope	−3.47	−3.246	−3.79	−3.64	−3.31	−3.20	−3.51	−3.19	−2.56	−3.68	−3.19	−2.95	−3.71	−3.36
Y intercept	38.07	37.09	38.55	41.18	36.56	38.40	36.88	37.47	36.17	36.65	36.83	37.51	37.52	37.32
**Clade II 2022**														
Slope	−2.999	−3.109	−3.165	−2.558	−3.295	−3.455	−3.31	−3.34	−3.82	−3.67	−3.18	−2.95	−3.70	−3.36
Y intercept	37.08	36.41	37.87	36.17	36.49	37.66	37.17	36.61	39.38	36.65	36.83	37.51	37.52	37.32
**Clade I**														
Slope	−3.18	−3.99	ND^a^	−3.83	−3.26	−3.08	−3.63	−2.91	−2.37	−3.35	−3.13	−3.59	−3.21	−2.90
Y intercept	38.9	40.3	ND^a^	42.12	38.37	39.94	39.1	38.85	37.33	38.07	38.95	40.5	38.67	37.65

MPXV: monkeypox virus; OPV: orthopoxvirus; ref: reference PCR; ND: not detected.

Slope and Y-intercept resulting from six fourfold dilutions of Clade II 2012 and Clade II 2022 and three eightfold dilutions of Clade I DNA.

^a^ The MPXV Clade II-specific PCR does not detect DNA from MPXV Clade I.

We observed small variations in Cq values per sample but in general, all kits resulted in comparable standard curves, particularly for both Clade II viruses. 

## Discussion

For roughly 50 years, human monkeypox was rarely detected outside of central and western Africa [8], with one larger outbreak in the United States (US) in 2003 linked to rodents imported from Ghana [9]. Individual cases have been reported in non-endemic regions, such as the United Kingdom, the US, Singapore and Israel, with links to travel to endemic countries, but with limited onward transmissions. 

Proper sampling of monkeypox lesions generally results in low Cq values (high virus loads) [10], therefore all 11 evaluated PCR kits are probably suitable for diagnosis of MPXV in skin lesions. However, poor sampling may impact the test accuracy; inclusion of endogenous human positive controls in the kits may help understand if inadequate sampling occurred in case of a negative result in a suspected patient. Further, sampling at an alternative location may require more sensitive PCR detection to ensure accurate diagnosis, as the viral kinetics may vary.

In some manuals, the limit of detection is given as copies/mL which is not an optimal metric for certain sample types, such as crusts and dry swabs. An exact quantification of virus DNA in lesions and other tissue samples is hampered by the lack of a reference standard, but quantification in primary poxvirus diagnostics is not of great relevance.

A potential limitation of the study is that we used only three viruses for specificity testing which are relevant for differential diagnostics, but the study is an evaluation and not a full validation.

## Conclusion

The 11 evaluated kits showed comparable and high sensitivity to detect Clade I and Clade II monkeypox virus DNA and were therefore suitable to identify a range of clinically relevant viral loads of MPXV DNA for diagnosis using properly sampled skin lesions [10]. Analytical sensitivity of the kits was generally high, detecting down to less than ca 5 ge/rxn (Cq = 36), and the limited specificity assessment showed that most assays were specific for MPXV or OPV, as per their intended design. It should be noted that the included kits and the many others coming to market are currently intended for research use only; it is still necessary to generate and disseminate data assessing clinical performance to ensure increased adoption of accurate kits that enable access to monkeypox diagnosis in communities which are most affected by the disease. 

## Supplementary Data

319000 Supplement
