# Supplementary material for: Evaluation of 11 commercially available PCR kits for the detection of monkeypox virus DNA, Berlin, July to September 2022
Source: Euro Surveill. 2022 Nov 10;27(45):2200816. doi: 10.2807/1560-7917.ES.2022.27.45.2200816 (PMC9650706; doi:10.2807/1560-7917.ES.2022.27.45.2200816)

This supplementary material is hosted by *Eurosurveillance* as supporting information alongside the article [Evaluation of 11 commercially available PCR kits for the detection of monkeypox virus DNA, Berlin, July-September 2022], on behalf of the authors, who remain responsible for the accuracy and appropriateness of the content. The same standards for ethics, copyright, attributions and permissions as for the article apply. Supplements are not edited by *Eurosurveillance* and the journal is not responsible for the maintenance of any links or email addresses provided therein.

Supplementary Table 1: Reaction components for PCRs used as reference for detection of monkeypox virus DNA

Reaction 1: Duplex OPV generic / KoMa			Reaction 2: MPXV generic		
Platinum *Taq* DNA-Polymerase, Invitrogen<br>Cat No 10966034			Platinum qPCR SuperMix-UDG, Invitrogen<br>Cat No 11730025		
Component	Stock concentration	Volume per reaction (µL)	Component	Stock concentration	Volume per reaction (µL)
PCR-grade water	-	9.80	PCR-grade water	-	4.00
10x PCR Buffer	10 x	2.50	SuperMix	-	12.50
$MgCl_2$	50 mM	2.00	$MgCl_2$	50 mM	1.00
dNTPs	2.5 mM	2.00	G2R_G F	10 mM	1.00
rpo F	10 µM	0.75	G2R_G R	10 µM	1.00
rpo R	10 µM	0.75	G2R_G probe	10 µM	0.50
rpo probe	10 µM	0.25			
KoMa F	10 µM	0.75			
KoMa R	10 µM	0.75			
KoMa probe	10 µM	0.25			
Platinum Taq Polymerase	10 x	0.20			
Reaction 3: MPXV clade II			Reaction 4: *MYC*		
Platinum qPCR SuperMix-UDG, Invitrogen<br>Cat No 11730025			Platinum qPCR SuperMix-UDG, Invitrogen<br>Cat No 11730025		
Component	Stock concentration	Volume per reaction (µL)	Component	Stock concentration	Volume per reaction (µL)
PCR-grade water	-	4.00	PCR-grade water	-	4.75
SuperMix	-	12.50	SuperMix	-	12.50
$MgCl_2$	50 mM	1.00	$MgCl_2$	50 mM	1.00
G2R_WA F	10 µM	1.00	c-myc F	10 µM	0.75
G2R_WA R	10 µM	1.00	c-myc R	10 µM	0.75
G2R_WA probe	10 µM	0.50	c-myc probe	10 µM	0.25

OPV: orthopoxviruses; MPXV: monkeypox virus

Supplementary Table 2: Reaction conditions for PCRs used as reference for detection of monkeypox virus DNA on BioRad CFX96[a]

**Temperature**	**Time (min:sec)**	**Number of cycles**
95 °C	05:00	1
95 °C	00:15	45
60°/62[b]°C	00:30	

[a] Other real-time PCR cyclers may require adapted thermoprofiles.
[b] For reaction 3.

Supplemental figure 1

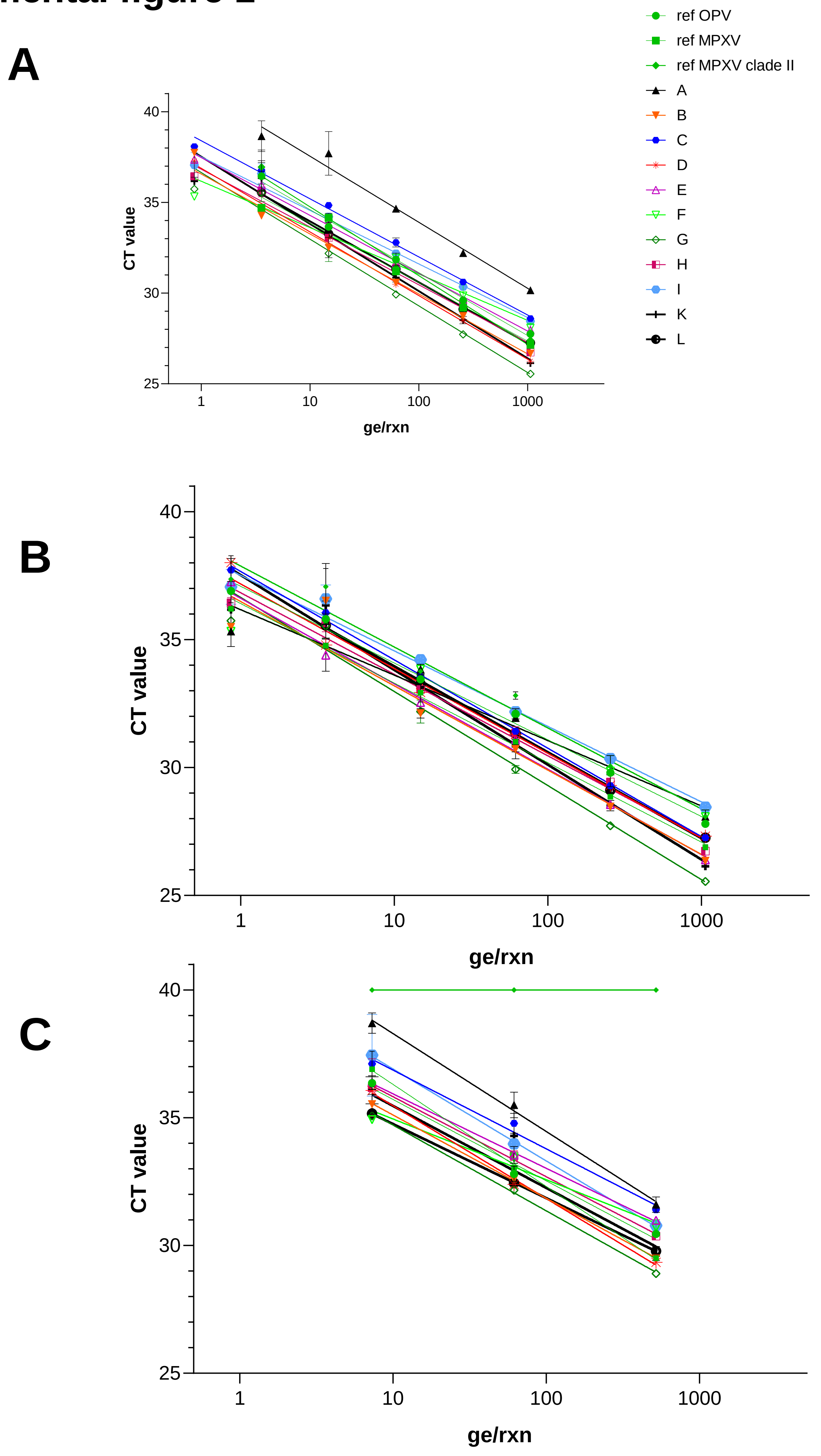

Supplement: Supplement [file 22-00816_MICHEL_Supplement.pdf]
